# Honokiol Inhibits Melanoma Growth by Targeting Keratin 18 *in vitro* and *in vivo*

**DOI:** 10.3389/fcell.2020.603472

**Published:** 2020-11-24

**Authors:** Tingting Liu, Hui Liu, Penglei Wang, Yamei Hu, Ran Yang, Fangfang Liu, Hong Gyum Kim, Zigang Dong, Kangdong Liu

**Affiliations:** ^1^Department of Pathophysiology, School of Basic Medical Sciences, Zhengzhou University, Zhengzhou, China; ^2^China-US (Henan) Hormel Cancer Institute, Zhengzhou, China; ^3^Henan Provincial Cooperative Innovation Center for Cancer Chemoprevention, Zhengzhou University, Zhengzhou, China; ^4^State Key Laboratory for the Prevention and Treatment of Esophageal Cancer, Zhengzhou University, Zhengzhou, China; ^5^Cancer Chemoprevention International Collaboration Laboratory, Zhengzhou, China

**Keywords:** honokiol, keratin18, degradation, ubiquitination, melanoma

## Abstract

Honokiol, a natural compound, derived from *Magnolia officinalis*, has been shown to have anti-cancer effect in several cancer types. However, the underlying molecular mechanism associated with its anti-cancer properties has not been fully elucidated. In the current study, we showed that honokiol inhibited the growth of melanoma cells in a dose and time-dependent manner. Mechanistically, it directly interacts with keratin 18 (KRT18) protein and induces its degradation through ubiquitination. Furthermore, the expression of KRT18 was found to be higher in melanoma tissues compared to the normal skin tissues. In addition, KRT18 overexpression significantly promoted melanoma cell proliferation and growth. Our results showed that honokiol treatment significantly decreased KRT18 protein level and suppressed the tumor growth in melanoma cell-derived xenograft mice models. Hence, KRT18 plays an oncogenic role in melanoma and honokiol can be an inhibitor for KRT18.

## Introduction

Melanoma as an aggressive cancer with poor prognosis is responsible for most number of skin cancer related deaths. According to the American Cancer Society, it is estimated that 1,00,350 new melanoma cases, and 6,850 related deaths will occur in the United States alone, in 2020 (American Cancer Society, 2020). Furthermore, the melanoma incidences continue to increase worldwide, and it is the fifth most common cancer in men and sixth most common cancer in women in the United States (Carr et al., 2020). However, it was estimated that the death rates remain to be stable, whereas, the annual cost of treating newly diagnosed melanoma cases to increase from $457 million in 2011 to $1.6 billion in 2030 (Guy et al., 2015). The risk factors contributing to melanoma remains largely unchanged, including sun exposure and ultraviolet light (UV). Substantial reductions in the melanoma incidences, mortalities, and treatment cost can be achieved by comprehensive interventions including reduced UV radiation exposure and increased sun protection. However, there is an urgent need to discover novel effective therapeutic targets and inhibitors to suppress the tumor progression and aid in the chemoprevention of melanoma.

As a type I cytokeratin of the intermediate filament family member of cytoskeleton, Keratin 18 (*KRT18*) plays an important role in various cellular processes, including maintaining the structural integrity of cytoplasm and mitochondria, and withstanding external stress (Weng et al., 2012). Together with its filament partner keratin 8 (*KRT8*), it is expressed in single layer epithelial tissues of the body. KRT18 has a potential biomarker effect in chronic kidney disease, non-alcoholic fatty liver disease, hepatitis C virus patients and acute intestinal graft versus host disease (Roth et al., 2011; Sauer et al., 2018; Darweesh et al., 2019). Recently, it was reported that KRT18 acts as an oncogene and is aberrantly expressed in several human malignancies as well as correlates with clinical progression and prognosis (Zhang et al., 2019; Wang et al., 2020; Yin et al., 2020). However, the biological role of KRT18 is seldom reported in melanoma.

Honokiol (C_18_H_18_O_2_) is a natural biphenolic compound extracted from the leaves and barks of *Magnolia officinalis*, and is widely used in traditional Chinese medicine. Honokiol has been reported to have several pharmacological effects, including anti-inflammation, anti-aging, anti-bacterial, neuroprotective, anti-oxidant, and anti-cancer effects (Sarrica et al., 2018; Ong et al., 2019). The anti-cancer activity of honokiol has been studied in various tumors (Fried and Arbiser, 2009; Rauf et al., 2018). Furthermore, studies have shown that honokiol can regulate various molecular targets, including activation of pro-apoptotic factors, suppression of anti-apoptotic proteins and different transcription factors, down-regulation of various enzymes, and inhibition of chemokines, cell surface adhesion molecules, cell cycle proteins, and kinase activity (Banik et al., 2019; Lee et al., 2019; Wang et al., 2019). However, the exact role of honokiol in melanoma and the associated molecular mechanism remains largely unknown. In the current study, we investigated the role of honokiol in the suppression of melanoma growth, *in vitro* and *in vivo*. Mechanistically, honokiol directly binds to KRT18 protein and decreases its stability through ubiquitination. Our study demonstrates that KRT18 plays an oncogenic role in melanoma and promotes melanoma growth, while honokiol can be an inhibitor for KRT18.

## Materials and Methods

### Chemicals and Reagents

Honokiol (CAS: 35354-74-6, purity >98%) was purchased from Dalian Meilun Biotechnology Co., Ltd., China. Gentamicin sulfate, puromycin, 100X penicillin-streptomycin liquid and trypsin-EDTA solution were obtained from Solarbio (Beijing, China). Polybrene, used for viral infection of cells was obtained from Merck (Darmstadt, Germany). Minimal essential medium with Earle’s balanced salt solution (MEM/EBSS) was purchased from Hyclone (Utah, United States). Keratinocyte serum-free media was obtained from Invitrogen (Carlsbad, CA, United States). Fetal Bovine Serum (FBS) and Dulbecco’s modified Eagle’s medium (DMEM; high glucose) were obtained from Biological Industries (Beit-Haemek, Israel). Human KRT18 protein was purchased from OriGene (Maryland, United States). The primary antibodies against KRT18 (ab668) and Ki-67 (ab16667) were obtained from Abcam (Cambridge, United Kingdom), whereas, HA-tag (26183) and Flag-tag (F1804) antibodies were from Thermo Fisher Scientific (Waltham, MA, United States) and Sigma-Aldrich (St. Louis, MO, United States), respectively. GAPDH (TA-08) was from ZSGB-Bio Inc., Beijing, China. The goat anti-rabbit IgG-HRP (sc-2004) and goat anti-mouse IgG-HRP (sc-2005) secondary antibodies were purchased from Santa Cruz Biotechnology (Santa Cruz, CA, United States).

### Cell Culture

SK-MEL-2, SK-MEL-5, SK-MEL-28, and MM200 human melanoma cell lines and HEK293T human embryonic kidney cell line were purchased from American Type Culture Collection (ATCC; Manassas, VA, United States). All cells were cytogenetically tested and authenticated before being frozen. SK-MEL-2, SK-MEL5, and SK-MEL-28 were cultured in MEM/EBSS with 10% (v/v) FBS and 1% (v/v) penicillin-streptomycin liquid, whereas MM200 and HEK293T cells were cultured in DMEM supplemented with 10% (v/v) FBS and 1% (v/v) penicillin-streptomycin liquid. N-TERT human skin keratinocytes cell was purchased from the Harvard Skin Disease Research Center and cultured in keratinocyte serum-free media supplemented with 25 μg/mL BPE, 0.4 mM Ca^2+^, 0.2 ng/mL EGF, 5 mM L-glutamine and 1% (v/v) penicillin-streptomycin liquid. Cells were maintained at 37°C in a humidified environment with 5% CO_2_.

### Anchorage-Independent Cell Growth Assay and Cell Proliferation Assay

The plates contained 3 mL of bottom agar and 1 mL of top agar. The concentration of honokiol was maintained same between the bottom (MEM/10, FBS/0.5% agar) and the top agar (MEM/10, FBS/0.33% agar). The cells (8 × 10^3^ cells/well) were plated into the top agar of each well and incubated at 37°C and 5% CO_2_ for 2–3 weeks. Images were captured using the microscope and results were analyzed by Image J software.

SK-MEL-5 or SK-MEL28 cells were seeded into 96-well plates for 24 h. After seeding for 24 h, the cells were treated with different concentrations of honokiol (0, 10, 20, 30, 40, or 50 μM) for 24, 48, 72, or 96 h. Following the treatment, cells were incubated with 20 μL of 3-[4, 5-dimethylthiazol-2-yl]-2, 5 diphenyltetrazolium bromide (MTT) solution (5 mg/mL) at 37°C and 5% CO_2_ for 2 h. Then, the medium was discarded followed by addition of 150 μL of DMSO, and the absorbance was measured at 490 nm with Thermo Scientific Multiskan Sky plate-reader (Thermo Fisher Scientific, Waltham, MA, United States).

### Western Blot Analysis

Cells were washed twice with ice-cold phosphate-buffered saline and then disrupted using radio immunoprecipitation assay (RIPA) lysis buffer (with phosphatase and protease inhibitors). Cell lysates were centrifuged at 12,000 × *g* for 15 min at 4°C and the supernatant fractions were collected. Bicinchoninic acid (BCA) protein assay kit (Solarbio, Beijing, China) was used for the quantification of total protein. Cell lysates (20 – 40 μg) were separated by SDS-PAGE and the bands were transferred to polyvinylidene fluoride membranes (Millipore, Billerica, MA, United States). The membranes were then blocked with 5% skim milk in TBS containing 0.1% Tween-20 (TBST) followed by overnight incubation with antibodies against KRT18 (1:1000) or GAPDH (1:2000) at 4°C. The blots were then incubated with goat anti-mouse/rabbit IgG-HRP secondary antibodies for 1 h at room temperature (RT). Following the incubation, protein bands were visualized using the enhanced chemiluminescence reagent (Millipore, Billerica, MA, United States) and Amersham Imager 600 Ultra-sensitive multi-function imager (GE Healthcare, America).

### *In vitro* Pull-Down Assay of KRT18

Sepharose^TM^ 4B-beads (GE Healthcare, Uppsala, Sweden) coupled with honokiol were incubated with recombinant KRT18 protein at 4°C, overnight, whereas, Sepharose^TM^ beads without honokiol were used as an inactive control. Further, to investigate the binding of KRT18 and honokiol, *in vitro*, whole cell lysates of SK-MEL-5, SK-MEL-28, and KRT18 overexpressed HEK293T were incubated with Sepharose^TM^ 4B-beads-honokiol at 4°C, overnight. Then, the beads were washed 3–5 times, each for 5 min with RIPA buffer and the eluted proteins were analyzed by western blotting.

### Immunohistochemistry

Malignant melanoma tissue chip (ME1002a) obtained from Xi’an Alenabio Technology Co., Ltd., China was used for IHC analysis. After dewaxing, hydration and antigen repair, the tissue chip was stained using SP kit (streptavidin-biotin detection system; ZSGB-Bio, Beijing, China). Briefly, tissue sections were incubated with 3% H_2_O_2_ for 5 min, blocked with goat serum for 1 h at RT, and incubated with primary antibody against KRT18 (1:200) at 4°C, overnight. The tissue sections were then incubated with secondary antibodies at RT for 2 h, followed by incubation with horseradish peroxidase enzyme-labeled streptavidin working solution for 30 min at RT. DAB (3,3-diaminobenzidine) staining, hematoxylin counterstaining, and then neutral resin mounting were performed. For the detection of Ki-67, the tumor tissues fixed in 4% formaldehyde were embedded in paraffin, sectioned and then stained by IHC. The method was same with KRT18 detection and the primary antibody was diluted 1:200 with PBS. The tissue sections were observed under the microscope and the results were analyzed using the Aperio Image Scope software (v11.1.2.752).

### Lentiviral Construction and Infection

*pLKO.1* or *pLKO.1*-shKRT18 and lentivirus packaging plasmids (*psPAX2* and *pMD2.G*) were transfected into HEK293T cells with Simple-Fect Transfection Reagent (Signaling Dawn Biotech, Hubei, China). Collect the virus at 24 or 48 h after changing the fresh medium. SK-MEL-5 and SK-MEL-28 melanoma cells were infected with KRT18 knockdown viral particles together with 8 μg/mL of polybrene (Merck, Darmstadt, Germany), and the transfected clones were selected with 5 μg/mL puromycin.

### Co-immunoprecipitation Assay

The vectors, *pRK5*-HA-ubiquitin and *pCDNA3.1*-flag-KRT18 were co-transfected into HEK293T cells with Simple-Fect Transfection Reagent, and the co-transfected cells were then treated with honokiol. Whole cell lysates were prepared and incubated with flag antibody to immunoprecipitate HA-ubiquitin complex; HA antibody was used to determine the ubiquitination level of KRT18 by western blotting.

### Melanoma Cells Derived Xenograft Mice Model

The animal experiments were performed according to the guidelines approved by the Zhengzhou University Institutional Animal Care and Use Committee. Female CB17 severe combined immunodeficient mice, aged 5–6 weeks were purchased from Charles River (Beijing, China) were divided into 3 groups (*n* = 8 mice per group) as follows: vehicle control, 30 mg/kg or 50 mg/kg honokiol treatment group. SK-MEL-5 or SK-MEL-28 cells (1.5 × 10^6^ cells) were mixed with an equal volume of Matrigel, and 100 μL of the mixture was injected subcutaneously into the mice. The mice were then treated with vehicle control (1% carboxymethylcellulose sodium in normal saline) or different concentrations of honokiol through intraperitoneal injection. The tumor size was measured twice a week and tumor volume was calculated based on the following formula: $\text{Tumor}\,\text{volume}=\text{length}\times \text{width}\times \text{height}\times \frac{\pi }{6}$. At the end of the experiment, the mice were euthanized with CO_2_ inhalation and the tumors were immediately harvested. For subsequent studies, the tumor tissues were fixed in 4% formaldehyde for IHC analysis, and the remaining tumor tissues were quickly frozen in liquid nitrogen and stored at −80°C for western blot analysis.

### Statistical Analysis

Each experiment was performed in triplicate and the quantitative data are represented as mean ± standard deviation (SD). Significance between the groups was determined by non-parametric test. A *p*-value of <0.05 was considered statistically significant.

## Results

### Honokiol Inhibits Melanoma Cell Growth *in vitro*

**FIGURE 1:**
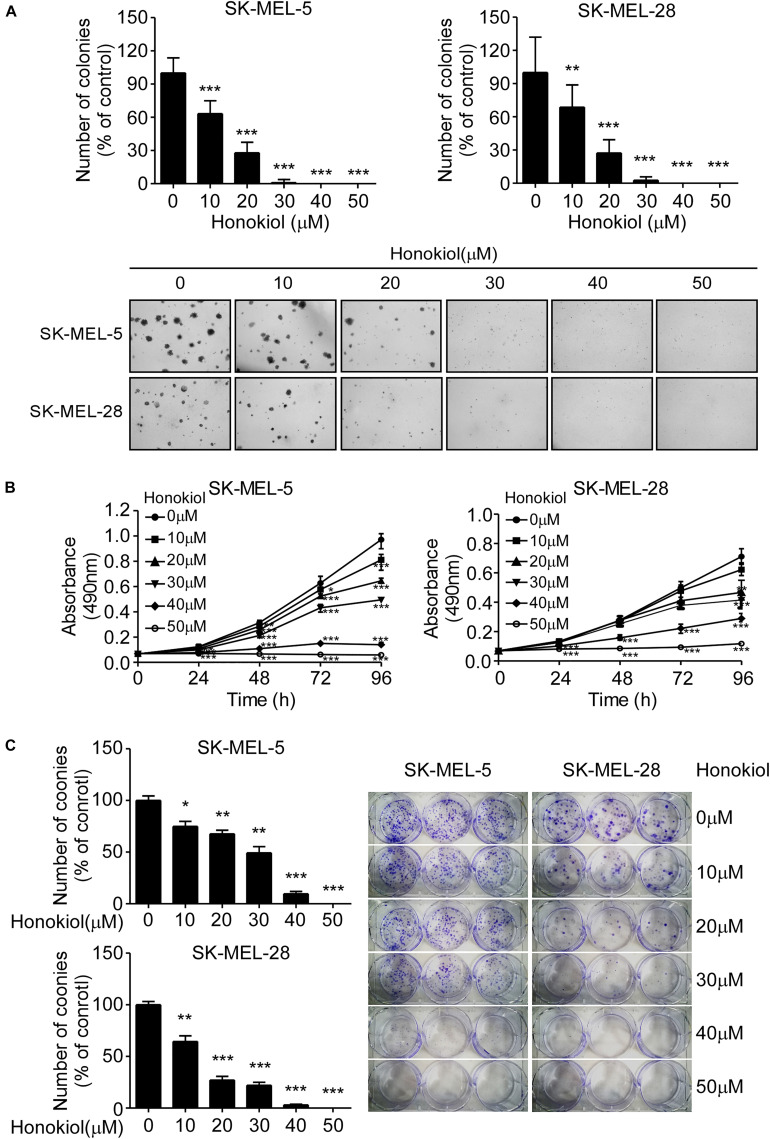
Honokiol significantly inhibits the growth of melanoma cells. **(A)** Effect of honokiol on anchorage-independent growth of melanoma cells. Clone count (above); Representative images of anchorage-independent cell growth results (below). **(B)** Effect of different honokiol concentration on proliferation of melanoma cells, as assessed by MTT assay. **(C)** Effect of honokiol on colony formation potential of melanoma cells, as assessed by plate colony formation assay. Clone number (left); Representative images (right). The asterisks indicate a significant difference between honokiol treatment and untreated control (*p < 0.05, **p < 0.01, and ***p < 0.001).

To evaluate the effect of honokiol on melanoma cell growth, SK-MEL-5 and SK-MEL-28 cells were treated with different concentrations of honokiol (0, 10, 20, 30, 40, or 50 μM). The results showed that honokiol significantly suppressed the anchorage-independent growth of the melanoma cells (Figure 1A). Additionally, treatment of the melanoma cells with different concentrations of honokiol for 24, 48, 72, or 96 h indicated a dose-and time-dependent manner inhibition in cell proliferation (Figure 1B). Furthermore, honokiol treatment significantly inhibited the colony forming ability of melanoma cells (Figure 1C). Together, these results indicate that honokiol significantly inhibits the growth of melanoma cells in a dose-and time dependent manner.

### KRT18 Is a Potential Target of Honokiol in Melanoma

**FIGURE 2:**
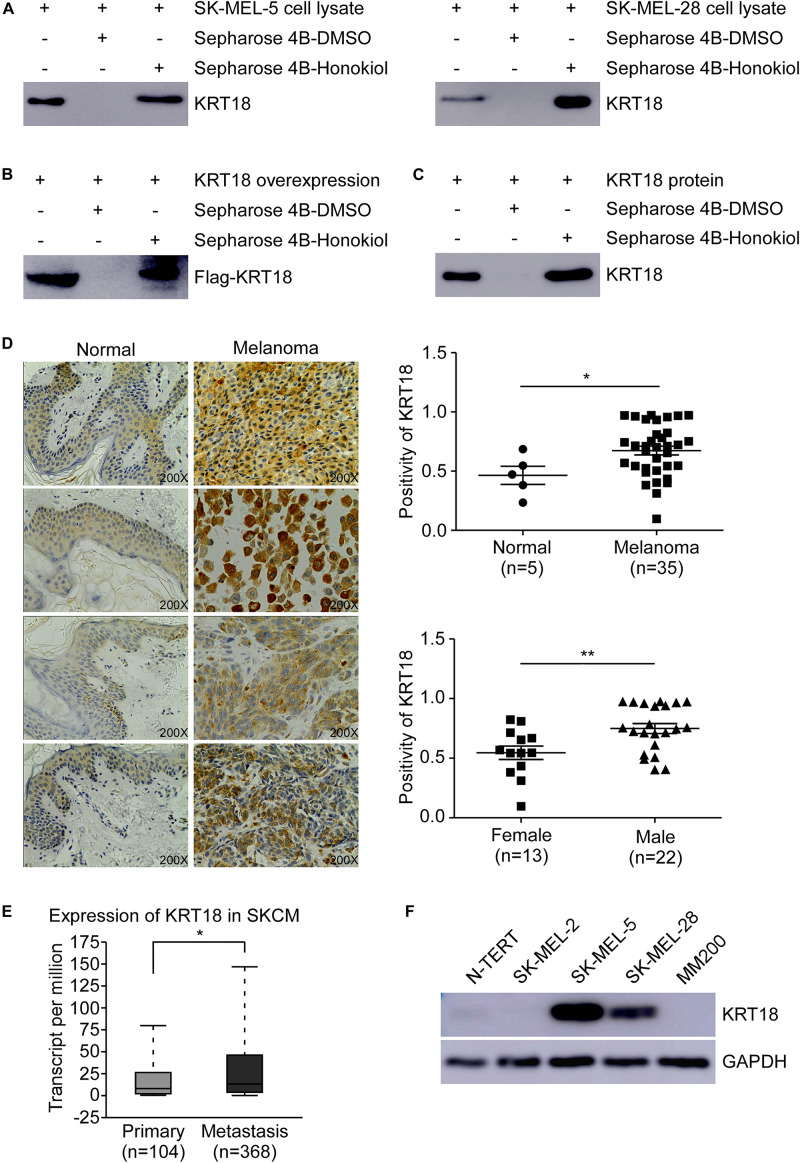
Honokiol directly binds to KRT18, which is highly expressed in some melanoma cells and tissues. Honokiol directly binds to KRT18 **(A)** in melanoma cell lysate, **(B)** exogenously and **(C)** recombinant protein. Proteins were pulled down by Sepharose 4B-honokiol beads or with Sepharose 4B beads and then analyzed by western blotting. **(D)** Representative images of Immunohistochemical analysis of the melanoma tissue chip (left) and bar graphs showing the number of KRT18 stained cells by IHC staining through image scope (right). KRT18 protein level between normal skin and melanoma tissues (right, above) as well as between male and female melanoma tissues (right, down). **(E)** KRT18 expression between primary and metastatic melanoma tissues from TCGA database (SKCM: Skin Cutaneous Melanoma). **(F)** KRT18 protein level between melanoma cell lines and normal skin cell line. The asterisks indicate a significant difference (*p < 0.05 and **p < 0.01 compared to control.

To identify the potential molecular target of honokiol in melanoma, we performed pull-down assay and mass spectrometry. Honokiol could pull down KRT18, suggesting KRT18 to be a potential target of honokiol in melanoma. Hence, to verify the interaction between KRT18 and honokiol in melanoma, we performed *in vitro* pull-down assay by incubating SK-MEL-5 or SK-MEL-28 cell lysates with honokiol. The results showed that honokiol could directly bind to KRT18 (Figure 2A). The binding of honokiol to KRT18 was further confirmed by pull down assays of exogenous and recombinant KRT18 (Figures 2B,C). Furthermore, IHC staining using a melanoma tissue array, showed that KRT18 is significantly overexpressed in the melanoma tissues compared to the normal skin tissues (Figure 2D). Interestingly, the expression of KRT18 was comparatively higher in the melanoma tissues of male patients than their female counterparts (Figure 2D). In addition, KRT18 mRNA level was found to be higher in the metastasis melanoma tissues than in the primary melanoma tissues in the TCGA database (Figure 2E). Furthermore, western blotting results showed higher expression of KRT18 in some melanoma cell lines than that in the normal skin cell (Figure 2F). Overall, our results indicate that KRT18 is highly expressed in the melanoma tissues, at both mRNA and protein levels.

### KRT18 Promotes Melanoma Cell Growth

**FIGURE 3:**
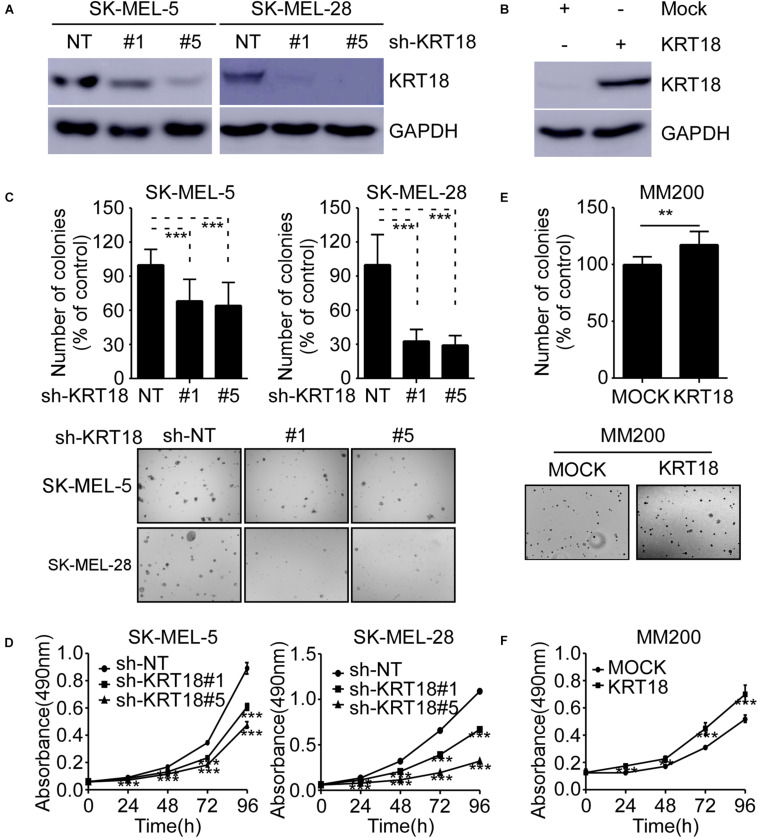
KRT18 promotes melanoma cell growth. **(A)** Western blot analysis showing KRT18 expression in SK-MEL-5 and SK-MEL-28 cells stably transfected with sh-NT or sh-KRT18. **(B)** Western blot analysis showing KRT18 expression in MM200 cells overexpressed KRT18. **(C)** Effect of KRT18 knockdown on anchorage-independent growth of SK-MEL-5 and SK-MEL-28 melanoma cells was evaluated by soft agar assay. Clone count (above); Representative images (below). **(D)** Effect of KRT18 knockdown on the proliferation of SK-MEL-5 and SK-MEL-28 melanoma cells was evaluated at 0, 24, 48, 72, or 96 h by MTT assay. **(E)** Effect of KRT18 overexpression on anchorage-independent growth of MM200 melanoma cell was evaluated by soft agar assay. **(F)** Effect of KRT18 overexpression on the proliferation of MM200 melanoma cells was evaluated at 0, 24, 48, 72, or 96 h by MTT assay. The asterisk (**p < 0.01 and ***p < 0.001) indicates a significant difference compared to the control group.

To investigate the role of KRT18 in melanoma, we knocked-down or overexpressed KRT18 in melanoma cells. The efficacy of knockdown or overexpression experiments were verified by western blotting (Figures 3A,B). The cell viability after KRT18 knockdown or overexpression was determined by anchorage-independent growth assay and MTT assay. The results showed that knocking down of KRT18 significantly inhibited both cell proliferation and anchorage-independent colony formation of SK-MEL-5 and SK-MEL-28 melanoma cells (Figures 3C,D). On the contrary, overexpression of KRT18 in MM200 cells promoted cell proliferation and growth (Figures 3E,F). These results indicate that KRT18 can promote melanoma growth.

### Honokiol Inhibits Melanoma Cell Growth by Targeting KRT18

**FIGURE 4:**
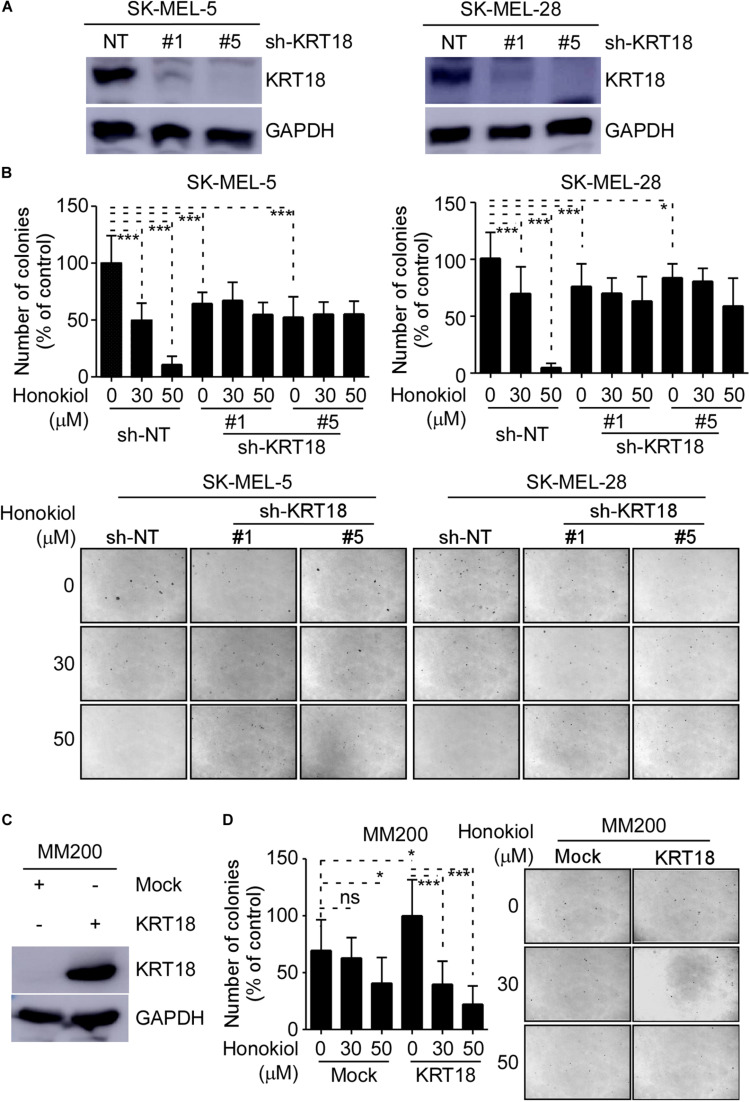
Inhibitory effect of honokiol on melanoma cell growth is dependent upon KRT18 protein level. **(A)** Western blot analysis showing KRT18 expression in SK-MEL-5 and SK-MEL-28 cells stably transfected with sh-NT or sh-KRT18. **(B)** Effect of KRT18 knockdown on anchorage-independent growth of honokiol treated SK-MEL-5 and SK-MEL-28 melanoma cells was evaluated by soft agar assay. Clone count (above); Representative images (below). **(C)** KRT18 protein level after KRT18 overexpression in MM200 melanoma cell. **(D)** Effect of KRT18 overexpression on anchorage-independent growth of honokiol treated MM200 melanoma cells was evaluated by soft agar assay. Clone count (left); Representative images (right). The asterisks (*p < 0.05, ***p < 0.001) indicate a significant difference in treated cells compared to the control cells.

We evaluated the effects of honokiol on melanoma growth after knocking-down or overexpressing KRT18 in melanoma cells and then treating them with honokiol. The KRT18 protein levels following KRT18 knockdown or overexpression were evaluated by western blotting (Figures 4A,C). Furthermore, melanoma cells with KRT18 stable knockdown or overexpression were treated with 0, 30 or 50 μM honokiol. The anchorage-independent colony forming assay results showed that the growth suppressing effects of honokiol was lower in cells with KRT18 knockdown, while it was higher in the cells with KRT18 overexpression (Figures 4B,D). Together, these results suggest that the growth inhibitory effect of honokiol depends upon KRT18 protein level in melanoma.

### Honokiol Induces KRT18 Degradation Through Ubiquitination

**FIGURE 5:**
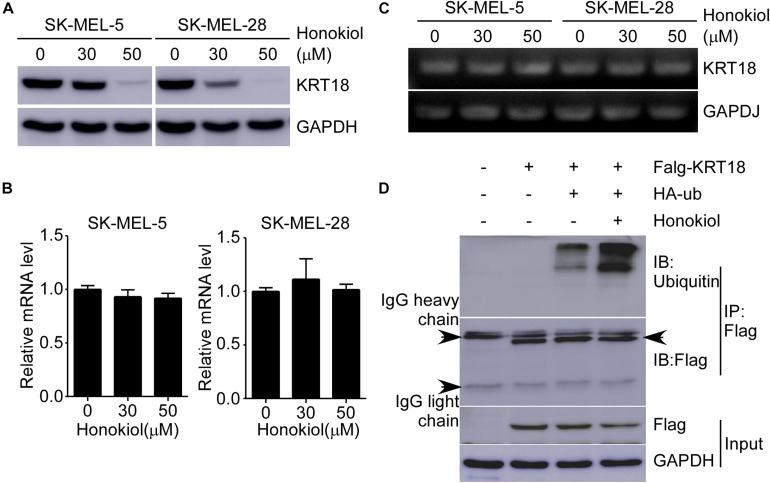
Honokiol down-regulates KRT18 protein levels by inducing its ubiquitination in melanoma cells. **(A)** Effect of honokiol on KRT18 protein expression in SK-MEL-5 or SK-MEL-28 melanoma cells. Cells were treated with 0, 30, or 50 μM of honokiol for 48 h and KRT18 protein level was evaluated by western blotting. **(B,C)** KRT18 mRNA level after honokiol treatment for 48 h was determined by RT-qPCR and agarose gel electrophoresis. **(D)** Immunoprecipitation of cell lysates from HEK293T cells transfected with exogenous flag-tagged KRT18 and HA-tagged ubiquitin extracts of exogenous flag was followed by immunodetection of exogenous ubiquitin and flag-KRT18. IP Flag: immunoprecipitation with a flag antibody.

To explore the detailed mechanism associated with the effect of honokiol on KRT18, first, we examined KRT18 protein level following honokiol treatment. The results showed that the KRT18 protein level significantly decreased after honokiol treatment in a dose-dependent manner (Figure 5A). However, RT-qPCR followed by agarose gel electrophoresis did not show any significant difference in KRT18 mRNA level upon honokiol treatment (Figures 5B,C), indicating that honokiol may regulate KRT18 at post-transcriptional level. Hence, we overexpressed HA-ubiquitin and Flag-KRT18 in HEK293T cells, and performed Flag-immunoprecipitation to determine the ubiquitination level of KRT18 in the treated cells. The results showed that honokiol induced KRT18 ubiquitination (Figure 5D). Thus, honokiol decreases KRT18 protein level by inducing its degradation through ubiquitination, in melanoma cells.

### Honokiol Inhibits Melanoma Cell Derived Xenograft Tumor Growth *in vivo*

**FIGURE 6:**
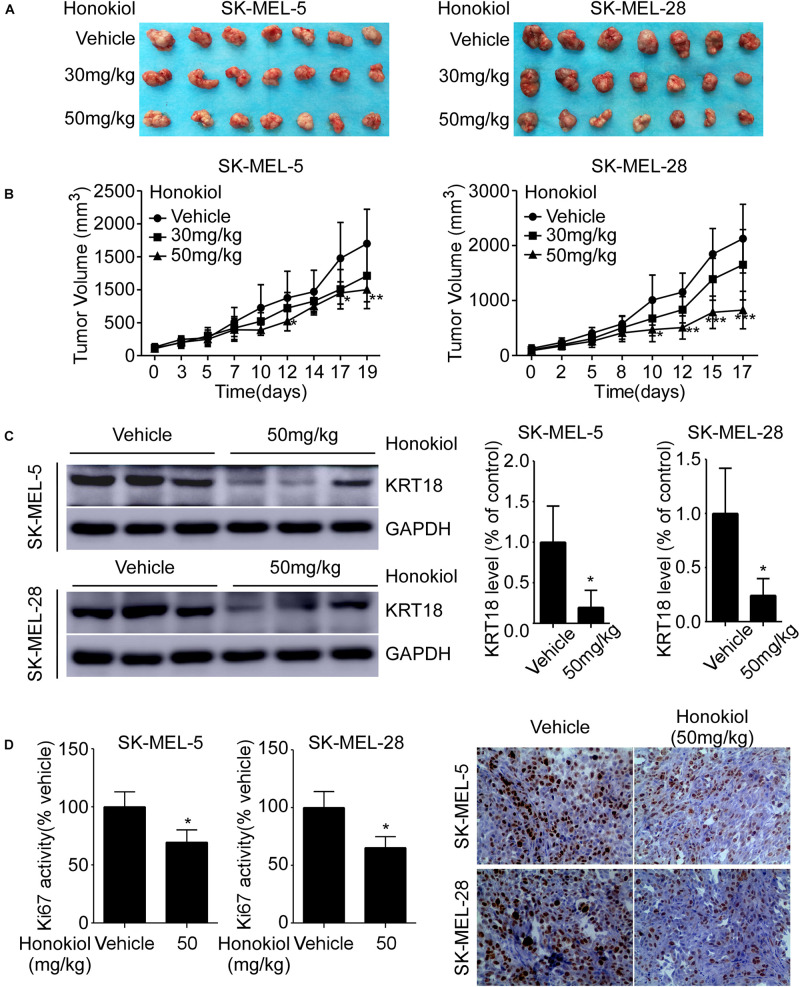
Honokiol inhibits melanoma cell-derived tumor growth *in vivo*. SK-MEL-5 or SK-MEL-28 melanoma cell-derived xenograft mice models were established and treated with varying concentrations of honokiol. **(A)** Morphology of tumors after honokiol treatment. **(B)** Effect of honokiol treatment on melanoma growth. Tumor size was measured twice a week and volume was calculated, as indicated in materials and methods. **(C)** KRT18 protein level in random three tissues of vehicle and 50 mg/kg honokiol groups, as detected by western blotting (left) followed by densitometric quantification (right). **(D)** Expression of Ki-67 in vehicle group or 50 mg/kg honokiol treatment group tumors, as assessed by IHC staining. Quantitation of Ki-67 (left); Representative images (right). Bar graphs quantifying the number of Ki-67 stained cells by IHC staining through image scope. The asterisks indicate a significant difference (*p < 0.05,**p < 0.01, ***p < 0.001) between the vehicle and honokiol treated groups.

To investigate the anti-cancer effect of honokiol *in vivo*, we established SK-MEL-5 or SK-MEL-28 cell-derived xenograft mice models. The results showed that the tumor volume was significantly decreased after honokiol treatment for 20 days (Figures 6A,B). Western blotting showed that honokiol treatment significantly decreased KRT18 protein level in tumor tissues (Figure 6C). Furthermore, IHC staining showed that the expression of tumor proliferation marker, Ki-67 significantly decreased in the honokiol treatment group compared to the vehicle group (Figure 6D). These results elucidate that the anti-cancer effect of honokiol in melanoma is mediated by reducing the level of KRT18 protein.

## Discussion

Melanoma continues to be a cancer with high incidences and death rates. However, there is no effective therapeutic target or inhibitor for melanoma treatment. In the current study, we found that honokiol can inhibit melanoma cell proliferation and growth, *in vitro*. Honokiol is a natural compound extracted from *M. officinalis*, and is widely used in traditional Chinese medicine. Furthermore, its anti-cancer effect has been reported in various cancer types. Our results showed that honokiol can exert its anti-cancer effect by binding to KRT18.

KRT18 is one of the keratins, also known as cytokeratins (KRTs), the intermediate filament forming proteins in epithelial cells (Cheng et al., 2019). The keratins are not only extensively used as tumor markers in cancer diagnosis, including in the detection of circulating tumor cells in carcinoma patients (Werner et al., 2019), but also their role in tumorigenesis and metastasis has gradually been valued (Dmello et al., 2019; Sharma et al., 2019). Multiple studies have reported that KRT18 can be used both as a biomarker and regulator in many diseases, including cancers (Weng et al., 2012). Additionally, knockdown of KRT18 has been reported to decrease the migration, and growth of cancer cells, and increase their sensitivity to chemotherapy, in non-small cell lung cancer, esophageal squamous cell carcinoma and renal cell carcinoma, suggesting that KRT18 has oncogenic potential in tumorigenesis (Ulukaya et al., 2007; Zhang et al., 2016; Yin et al., 2020). Conversely, KRT18 was reported to suppress tumor aggressiveness and paclitaxel-resistance in paclitaxel-resistant prostate and breast cancers (Fortier et al., 2013; Yin et al., 2016). In melanoma, a significant increase in the KRT18 mRNA level and its prognostic significance has been reported (Wang et al., 2004; Moll et al., 2008; Chen et al., 2009). However, the functional role of KRT18 in melanoma remains unclear. Our results showed higher expression KRT18 protein in melanoma compared to normal skin tissues. Furthermore, knockdown of KRT18 significantly decreased the proliferation of melanoma cells and their colony forming ability, indicating the oncogenic role of KRT18 in melanoma progression. Additionally, our results showed that the efficacy of honokiol was decreased after KRT18 knockdown, while increase with KRT18 overexpression, suggesting that KRT18 is targeted by honokiol. Further experiments revealed that honokiol induced KRT18 degradation via inducing its ubiquitination. The findings were further verified using melanoma cell-derived xenograft mice models.

In summary, our study revealed that honokiol inhibits melanoma growth both *in vitro* and *in vivo* by targeting KRT18. Mechanistically, honokiol binds to KRT18 inducing its degradation by ubiquitination. Hence, KRT18 play an oncogenic role in melanoma and honokiol can effectively inhibit the growth of melanoma. Furthermore, the current study not only elucidated the anti-melanoma effect of honokiol, but also provided a detailed molecular mechanism associated with its inhibitory effect on KRT18. Thus, our study will pave new ways for identifying melanoma targeted therapies in the foreseeable future.

## Data Availability Statement

The raw data supporting the conclusions of this article will be made available by the authors, without undue reservation.

## Ethics Statement

The animal study was reviewed and approved by Ethics Committee of China-US (Henan) Hormel Cancer Institute, Zhengzhou University, Zhengzhou, Henan, China.

## Author Contributions

TL performed most of the experiments and analyzed the data. PW performed animal experiments. HL and TL wrote the manuscript. YH, RY, and FL supported the method and regent. HK, ZD, and KL designed the research and supervised all the study. All authors contributed to the article and approved the submitted version.

## Conflict of Interest

The authors declare that the research was conducted in the absence of any commercial or financial relationships that could be construed as a potential conflict of interest.
